# Delayed drainage versus autotransfusion drainage and routine drainage after total knee arthroplasty: a comparative study

**DOI:** 10.1186/1749-799X-8-39

**Published:** 2013-11-11

**Authors:** Yuan Zhang, Zhi-jun Li, Yong-fa Zheng, Shi-qing Feng, Hui Li

**Affiliations:** 1 Department of Orthopedics, Tianjin Medical University General Hospital, Tianjin 300052, People's Republic of China

**Keywords:** Total knee arthroplasty, Delayed drainage, Autotransfusion drainage, Routine drainage, Comparative study

## Abstract

**Background:**

The purpose of this research is to compare the clinical results of different drainage methods in total knee arthroplasty (TKA).

**Methods:**

This retrospective comparative study included 55 patients who accepted primary unilateral TKA between October 2010 and November 2012. The patients were classified according to the drainage method used: 25 patients in the autotransfusion drainage group, 12 patients in the delayed drainage group, and 18 patients in the routine drainage group. Otherwise, the same operative procedures and postoperative care were applied to all patients. The variables recorded included total amount of postoperative drainage (including intraoperative blood loss); cases of allogenic blood transfusion; body temperatures on postoperative days 1, 3, and 7; and pre- and postoperative hemoglobin level. Some other elements such as postoperative swelling, range of motion, and wound healing were also compared.

**Results:**

Patients who underwent autotransfusion were found to have an amount of drainage (799.2 ± 196.7 mL) significantly greater than that in the routine drainage group (666.1 ± 155.0 mL), which in turn was significantly greater than that in the delayed drainage group (381.7 ± 129.2 mL). The postoperative hemoglobin level in the delayed drainage group (91.5 ± 7.9 g/L) was similar to that in the autotransfusion group (92.0 ± 9.6 g/L), while that in the routine drainage group (81.3 ± 9.9 g/L) was significantly lower. The patients in the autotransfusion group were observed to have higher body temperatures than those in the other two groups. In the routine drainage group, eight cases accepted allogenic blood transfusion, and the percentage (44.4%) was significantly higher than that in the other two groups. There were no significant between-group differences in swelling, healing qualities, and range of motion.

**Conclusions:**

Delayed postoperative drainage may reduce blood loss and the chance of allogenic blood transfusion compared with routine drainage and may also reduce the chance of postoperative fever and extra costs compared with autotransfusion.

## Background

As China's population ages, total knee replacement (total knee arthroplasty, TKA) is becoming more widely applied. Postoperative blood loss from current TKA techniques results in a significant volume of drainage [1-3]. How to control prolonged bleeding after TKA has provoked much discussion among surgeons. Any reduction in bleeding after joint replacement or in the amount of allogeneic blood transfusion can contribute not only to recovery from postsurgery trauma and avoidance of adverse reactions to transfusion but also to patients' early functional exercise and the clinical conservation of blood [4].

There are now many controversies over different drainage methods after TKA. Routine drainage is usually considered to cause articular pressure, resulting in the consistent decrease of pressure and, in turn, an increase in postoperative bleeding. Some researchers hold that avoiding drainage altogether may reduce postoperative blood loss [5]; however, after taking into account the potential for infection, limb swelling, and joint pain caused by inner tension and other unfavorable factors, most surgeons still advocate placing a drainage tube at the joint. Some other studies also recommend using an autologous drainage blood reinfusion device postoperatively to reduce the need for postoperative allogeneic blood transfusion [6-8].

This study is intended to research the clinical effects about three drainage methods following TKA, namely, autotransfusion drainage, delayed drainage, and routine drainage. It aims to (1) evaluate the influence of different drainage methods on postoperative blood loss, allogeneic blood transfusion, joint function, and other clinical indicators; and (2) identify the best postoperative drainage method after TKA.

## Methods

### Patients

This study, conducted between October 2010 and November 2012, was non-randomized and retrospective. The ethics committee of Tianjin Medical University General Hospital reviewed and approved the study (the reference number for ethics approval is 1218, and the trial registration number is 1218). It included 55 cases diagnosed with end-stage osteoarthritis or rheumatoid arthritis and admitted to the orthopedic department of our hospital. All patients received primary unilateral TKA excluding severe malformations and ipsilateral reoperation. Before surgery, all patients were informed of benefits, risks, and possible additional treatment expenses associated with different postoperative drainage methods. Consequently, balancing advantages and disadvantages, patients and their families could choose a postoperative drainage method and signed informed consent.

The inclusion criteria for patients were (1) clear diagnosis with end-stage osteoarthritis or rheumatoid arthritis on the basis of medical history; clinical examination; laboratory tests; frontal, lateral, and standing full-length X-ray of the standard weight-bearing knee; (2) intention to undergo primary unilateral TKA; (3) age ranging from 55 to 75 years; and (4) no significant systemic contraindications to surgery.

The exclusion criteria were (1) bilateral TKA; (2) revision knee arthroplasty; (3) obvious anemia or coagulopathy before surgery; (4) despite normal coagulation laboratory tests, long-term use of anticoagulants such as warfarin, aspirin, or clopidogrel because of other diseases; (5) relatively severe joint deformity (flexion deformity >10°, knee varus deformity >15°) and potential extensive soft tissue release; (6) non-postoperative drainage or accidental disconnection of the drainage tube.

The patients were classified into three groups: 25 patients in the autotransfusion drainage group (including 9 men and 16 women, average age of 62.6 ± 6.8 years), 12 patients in the delayed drainage group (4 men, 8 women, 65.3 ± 7.2 years), and 18 patients in the routine drainage group (8 men and 10 women, 63.7 ± 6.1 years). The general data for patients in each group are shown in Table 1.

**Table 1:** The general condition of the patients

**Group**	**Age**	**Gender (male/female)**	**Operation length (min)**	**Disease (RA/OA)**	**Preoperative hemoglobin (g/L)**
Routine drainage (*n* = 18)	63.72 ± 6.10	8/10	87.50 ± 13.09	2/16	129.44 ± 7.22
Blood transfusion (*n* = 25)	62.56 ± 6.83	9/16	82.40 ± 12.64	3/22	130.36 ± 6.01
Delayed drainage (*n* = 18)	65.25 ± 7.22	4/8	86.50 ± 18.27	2/10	131.92 ± 5.66
Statistics	*F* = 0.666	*χ*^2^ = 0.469	*F* = 0.490	*χ*^2^ = 0.222	*F* = 0.544
*P* value	0.518	0.791	0.615	0.895	0.584

### Surgical techniques and postoperative treatment

All patients were under spinal anesthesia and underwent operations performed by the same group of surgeons led by our senior author (HL), who is experienced in TKA. A pneumatic tourniquet with a pressure of 380 mmHg was routinely applied and inflated after limb exsanguinations. An anterior incision was taken around the inner side of the patellar joint capsule to expose the joint. All patients were cemented with a Smith & Nephew G2 poststabilized prosthesis (GENESIS II Knee System, Smith & Nephew plc., London, UK). All posterior cruciate ligaments were removed after the osteotomies were accomplished. An intramedullary positioning method was used on the femoral side, while an extramedullary positioning method was used on the tibial side. Before the prosthesis was placed, bone grafts were used to fill the opening of the bone marrow cavity. After the placement of the prosthesis, the exposed cancellous bone surface was sealed by bone wax. All patients underwent reshaping of the patella, but some of them did not undergo resurfacing with denervation.

The drainage tube was placed by cutting off skin from the proximal lateral incision into the joint cavity. In the autotransfusion group, the outer end of the drainage tube was connected to an autotransfusion device (CBC II Consta VacTM, Stryker, Kalamazoo, MI, USA) so that under negative pressure, blood would flow through a 200-μm filter into the blood reservoir tank without any anticoagulant. All the cases needed to complete the reinfusion within 6 h from the beginning of collecting drainage blood [6]. In both the delayed drainage and the routine drainage groups, the drainage tube was connected to an ordinary sterile drainage bag. As for these latter two groups, the tourniquet was regularly loosened to allow electric coagulation to be applied on the active bleeding point before the wound was closed. After closure, the wound was covered by a thick sterile elastic bandage from the distal end of the calf to the proximal end of the thigh.

In the autotransfusion and routine drainage groups, the drainage tubes were kept open after surgery, while in the delayed drainage group, the drainage tubes were required to be clamped until 12 h later, and all the drainage tubes were supposed to be removed 24 h after surgery. All patients are required to elevate the affected limb after surgery and to apply ice packs to the surgical area. Subcutaneous injection of low molecular weight heparin calcium was administered 24 h after surgery to prevent the formation of venous thrombosis until 10 to 14 days later. Balancing the perioperative transfusion guidelines, patient complications, age, and other factors [9,10], the patients with hemoglobin <90 g/L receive allogeneic suspension of red blood cells on postoperative day 1. Meanwhile, all patients were given the same formulation of intravenous analgesia by patient-controlled pump.

### Data collection

Several key variables were documented such as the total drainage volume, including intraoperative bleeding when a tourniquet was opened to stop bleeding; allogeneic blood transfusion; hemoglobin levels before and after surgery; postoperative body temperature on days 1, 3, and 7; limb swelling (difference in the upper patellar diameter); wound healing; and joint motion on day 14.

### Statistical analysis

All data were processed by the statistical package SPSS 16.0 (SPSS Inc., USA). Inter-group comparisons of normally distributed data, reported as *x* ± *s*, used ANOVA. The count data were compared using the chi-square test with a significance level of *α* = 0.05.

## Results

### Baseline characteristics

Among the three groups, there was no significant difference in the age of patients, gender, or hemoglobin levels before surgery.

### Postoperative clinical indicators

As the results show in Table 2, the total postoperative drainage volume was much greater in the autotransfusion group (799.2 ± 196.7 mL) than those in the other two groups, while it was significantly less in the delayed drainage group (381.7 ± 129.2 mL) than that in the routine drainage group (666.1 ± 155.0 mL). The hemoglobin level on postoperative day 1 was not significant different between the autotransfusion group (92.0 ± 9.6 g/L) and the delayed drainage group (91.5 ± 7.9 g/L), but the routine drainage group (81.3 ± 9.9 g/L) was significantly different.

**Table 2:** The compartment of clinical indicators before and after surgery

**Group**	**Postoperative hemoglobin (g/L)**	**Drainage volume (mL)**	**Number of allogeneic blood transfusion**	**Maximum postoperative body temperature**			**Upper patellar diameter difference before and after surgery (cm)**	**Joint motion (degree)**
				**First day**	**Third day**	**Seventh day**		
Routine drainage (*n* = 18)	81.33 ± 9.86	666.1 ± 155.0	8	37.26 ± 0.55	36.89 ± 0.66	36.79 ± 0.53	2.56 ± 1.20	110.56 ± 11.36
Blood transfusion (*n* = 25)	91.96 ± 9.57**	799.2 ± 196.7*	4*	37.70 ± 0.53**	37. 31 ± 0.44*	37.09 ± 0.37*	2.68 ± 1.10	108.00 ± 11.27
Delayed drainage (*n* = 18)	91.50 ± 7.92**	381.7 ± 129.2**^△△^	2*	37.27 ± 0.41^△^	36.67 ± 0.47^△△^	36.67 ± 0.48^△△^	2.33 ± 0.89	107.50 ± 10.77
Statistics	*F* = 7.616	*F* = 24.167	*χ*^2^ = 5.087	*F* = 4.953	*F* = 6.834	*F* = 7.616	*F* = 0.406	*F* = 0.365
*P* value	0.001	0.000	0.079	0.011	0.002	0.001	0.669	0.696

Compared with routine drainage group, *p < 0.05, **p < 0.01. Compared with blood transfusion group △*p* < 0.05, △△*p* < 0.01.

Body temperature was much higher in the autotransfusion group than those in the other two groups on postoperative days 1, 3, and 7, which also meant significant difference. In the routine drainage group, the number of patients (eight patients, 44.4%) receiving allogeneic blood transfusion was greater than those of patients in the autologous blood transfusion group (four cases, 16.0%) and the delayed drainage group (two cases, 16.7%). There was no significant between-group difference in limb swelling, wound healing, and joint motion on postoperative day 14.

## Discussion

### Placement of drainage after TKA remains controversial

Generally speaking, pneumatic tourniquets were likely to be used during surgery. The major blood loss in knee replacement occurs mainly after releasing the tourniquet on the wounds. Usually, the blood loss measured by postoperative drainage within 24 h after surgery is called the obvious blood loss [11]. The remaining blood between the joint cavity and tissue, usually measured by limb swelling, is called the hidden bleeding.

Postoperative hemoglobin on day 1 may be an indicator of the total blood loss during surgery. Placement of drainage after TKA remains controversial because most joint surgeons insist on the necessity of drainage, [6,8,12] while some surgeons base their beliefs on some studies, saying that non-drainage will not affect long-term recovery or increase obvious blood loss [13,14].

During our study, postoperative drainage after TKA was not applied to three patients, and when the bedding of one patient was changed, the drainage tube was dislodged and not replaced afterwards. Because of the small number of non-drainage cases, they were not included in the comparison. However, some complications were found in four patients, such as obvious postoperative pain, limb swelling, and subcutaneous congestion, which delayed their rehabilitation. Accordingly, the safety and benefit of placing drainage after TKA still needs more evidence and case support.

### Drawbacks of autologous blood transfusion and routine drainage

Many surgeons favor postoperative autologous drainage blood transfusion to compensate for blood loss because it may reduce the chance that allogeneic blood transfusions will be needed [15,16]. In accordance with the operating instructions for autologous blood transfusion, the tourniquet to stop bleeding was not released, and the drainage tube was placed right after the incision was closed.

The study found that compared with the other two groups, the overall postoperative drainage volume was significantly increased. The possible reasons might be the following: on one hand, surgery cannot be targeted right to the potential bleeding sites; on the other, “cocktail” mixed injection for hemostasis around the joint cannot be applied to autologous blood transfusion patients.

Routine postoperative open drainage was clearly not satisfactory because the continuous drainage would always keep a low pressure in the articular cavity, but with the release of the tourniquet, beginning of painful stimuli, and regression of spinal anesthesia and copious fluid infusion, the blood pressure would bounce back to original preoperative levels or higher, which resulted in prolonged intra-articular bleeding from wound and drainage increase. Coupled with the use of an anticoagulant within 24 h after joint replacement, routine drainage would leave the postoperative bleeding more difficult to control.

The study found that compared with the delayed drainage group, the drainage volume of the routine drainage group was still significantly greater, regardless of the use of cocktail and other interventions assisting hemostasis. Although drainage blood was reinfused in the autologous blood transfusion group, the postoperative hemoglobin level was not significantly increased compared with that in the delayed drainage group (91.5 ± 7.9 g/L), which was identical to that in some recent reports [4]. Probably, the form and nature of retransfused red blood cells have been changed, so it was difficult to improve the patient's anemia. In addition, some elements from wound drainage blood such as fat particles, bone scraps, bone cement monomer, bacteria, free hemoglobin, inflammatory cytokines, coagulation factors, and fibrin degradation products may cause embolism, thrombosis, renal dysfunction, and the risk for bacteremia after reinfusion [7,8].

The results showed that patients with drainage reinfusion of autologous blood tended to suffer high chances of high and prolonged fever compared with the routine drainage group and the delayed drainage group, which had significant differences, but it may also be caused by the blood particles and other pyrogens in the unwashed reinfusion blood.

### Delayed drainage after TKA reduced drainage volume

The basic idea of non-drainage knee replacement is to tightly suture the incision and bandage it with proper pressure, which can make a small space within the joint cavity so as to maintain a certain pressure to reduce postoperative bleeding [17]. Delayed drainage makes a comprehensive use of this principle. To be specific, in the early postoperative period, the drainage tube is closed right after the surgery and opened from time to time until a few hours later. On the one hand, intra-articular pressure is maintained to stop bleeding from the wound so as to ensure no significant active bleeding within the joint before the application of anticoagulants. On the other hand, the intra-articular residual blood needs to be drawn by opening the drainage tube, which not only can stop bleeding by pressure but will also prevent hematocele, reduce joint swelling and pain, and reduce the risk for subcutaneous ecchymosis and infection caused by postoperative hematoma. Since the clinical application of this method, there is no consensus about when delayed drainage should begin. Many researchers have proposed different delay options arranging from 1, 2, and 4 to even 24 h [18,19]. It is generally believed that 4-h delay or more is more appropriate, because it is considered that blood loss takes 37% of the total amount 2 h after knee replacement surgery, which can reach 55% after 4 h [12,20]. Another study showed that the main bleeding after TKA stays in the first 12 h and that the delayed opening of the drainage tube does not reduce the amount of bleeding [19]. At the design stage, a lot of reported proposals were tested until it was found that it was better to open the drainage tube 12 h after occlusion than opening it after 4.6 h. The study found that compared with the other two groups, the delayed drainage method can significantly reduce drainage volume. To be specific, the hemoglobin level was significantly higher than that in the routine drainage group on day 1, while the number of patients who needed allogeneic blood transfusions was reduced. Besides, no postoperative fever and joint swelling were found, and wound healing and joint motion showed no difference compared with those of the other two groups.

### Limitations of the study

Frankly, there are also some limitations to the study: the retrospective and non-randomized design, which may have introduced hidden bias; the small number of study samples; some question about the validity of the method of evaluating hidden blood loss; short-term follow-up; and the non-comprehensive analysis of the study results. Different drainage methods to control bleeding after TKA should be compared in a prospective randomized trial so as to obtain more reliable conclusions.

## Conclusions

This comparison of methods of drainage after TKA suggests that delayed drainage can reduce overt bleeding and the need for allogeneic blood transfusion relative to routine drainage, and can reduce postoperative fever relative to autologous blood reinfusion. Delayed drainage merits further study in a randomized trial.

## Competing interests

The authors declare that they have no competing interests.

## Authors' contributions

HL conceived of the study and participated in its design and coordination. HL, SQF, and YFZ designed and performed the experiments, and collected the data. YZ and ZJL wrote the manuscript. ZJL and HL edited the manuscript. All authors read and approved the final manuscript.
